# New Plant-Parasitic Nematode from the Mostly Mycophagous Genus *Bursaphelenchus* Discovered inside Figs in Japan

**DOI:** 10.1371/journal.pone.0099241

**Published:** 2014-06-18

**Authors:** Natsumi Kanzaki, Ryusei Tanaka, Robin M. Giblin-Davis, Kerrie A. Davies

**Affiliations:** 1 Department of Forest Microbiology, Forestry and Forest Products Research Institute, Tsukuba, Ibaraki, Japan; 2 Division of Parasitology, Faculty of Medicine, University of Miyazaki, Miyazaki, Miyazaki, Japan; 3 Fort Lauderdale Research and Education Center, University of Florida/IFAS, Davie, Florida, United States of America; 4 Centre for Evolutionary Biology and Biodiversity, School of Agriculture, Food and Wine, The University of Adelaide, Waite Campus, Glen Osmond, South Australia, Australia; James Hutton Institute, United Kingdom

## Abstract

A new nematode species, *Bursaphelenchus sycophilus* n. sp. is described. The species was found in syconia of a fig species, *Ficus variegata* during a field survey of fig-associated nematodes in Japan. Because it has a well-developed stylet and pharyngeal glands, the species is considered an obligate plant parasite, and is easily distinguished from all other fungal-feeding species in the genus based upon these characters. Although *B. sycophilus* n. sp. shares an important typological character, male spicule possessing a strongly recurved condylus, with the “*B. eremus* group” and the “*B. leoni* group” of the genus, it was inferred to be monophyletic with the “*B. fungivorus* group”. The uniquely shaped stylet and well-developed pharyngeal glands is reminiscent of the fig-floret parasitic but paraphyletic assemblage of “*Schistonchus*”. Thus, these morphological characters appear to be an extreme example of convergent evolution in the nematode family, Aphelenchoididae, inside figs. Other characters shared by the new species and its close relatives, i.e., lack of ventral P1 male genital papilla, female vulval flap, and papilla-shaped P4 genital papillae in males, corroborate the molecular phylogenetic inference. The unique biological character of obligate plant parasitism and highly derived appearance of the ingestive organs of *Bursaphelenchus sycophilus* n. sp. expands our knowledge of the potential morphological, physiological and developmental plasticity of the genus *Bursaphelenchus*.

## Introduction

The fig syconium provides a unique and interesting habitat for microbes and microscopic invertebrates. Trees of the genus *Ficus* L. are pollinated by highly specialized fig wasps (Agaonidae). This fascinating relationship has become a model system for studying cospeciation and host switching [1]–[6]. In this relationship, female wasps carrying pollen enter the young fig through a small hole (ostiole) at the apex of the fig, pollinating it and laying eggs in individual female florets within the fig syconium. After pollination, the syconium develops and the ostiole swells shut during subsequent seed development. Fig wasp larvae feed within infested female florets (seed galls) and develop into winged female and wingless male adults. Males emerge first from their respective seed galls and bore holes into the seed galls housing females for mating access. They then bore exit holes through the syconial wall to allow female wasps carrying the pollen to exit [7], [8]. Thus, the fig syconium is often considered a closed environmental niche.

However, regardless of this apparently closed system, many different groups of phoretic and parasitic invertebrates, e.g., nematodes [9]–[12] and mites [11], [13], have been reported from figs and fig wasps. Further, the nematode genus *Parasitodiplogaster* Poinar, which parasitizes the fig wasps, has been examined as a model system of species radiation and the evolution of pathogenicity [10]. Nevertheless, because of the apparent ubiquity of such associations and the large number of *Ficus* species that occur worldwide (>700 species), the diversity of fig-associated nematodes (and mites) is far from being fully understood, and further intense surveys of diversity are needed.

During a field survey of fig and fig wasp-associated nematodes in Japan, a species of *Bursaphelenchus* was isolated from *F. variegata* Blume. Although two lethal plant pathogens are known [14], members of the genus *Bursaphelenchus* Fuchs is generally regarded as beetle (Coleoptera) or bee (Hymenoptera)-phoretic fungal feeders, and even the plant-parasitic species retain many of the morphological (functional) characters of fungal feeding in their ingestive organs [15]. However, the newly-discovered species appears to be morphologically adapted to being a plant parasite. The nematode is described herein as *B. sycophilus* n. sp., and its molecular phylogenetic status and morphological and biological characters are described and discussed.

## Materials and Methods

### Nematode isolation

**Figure 1:**
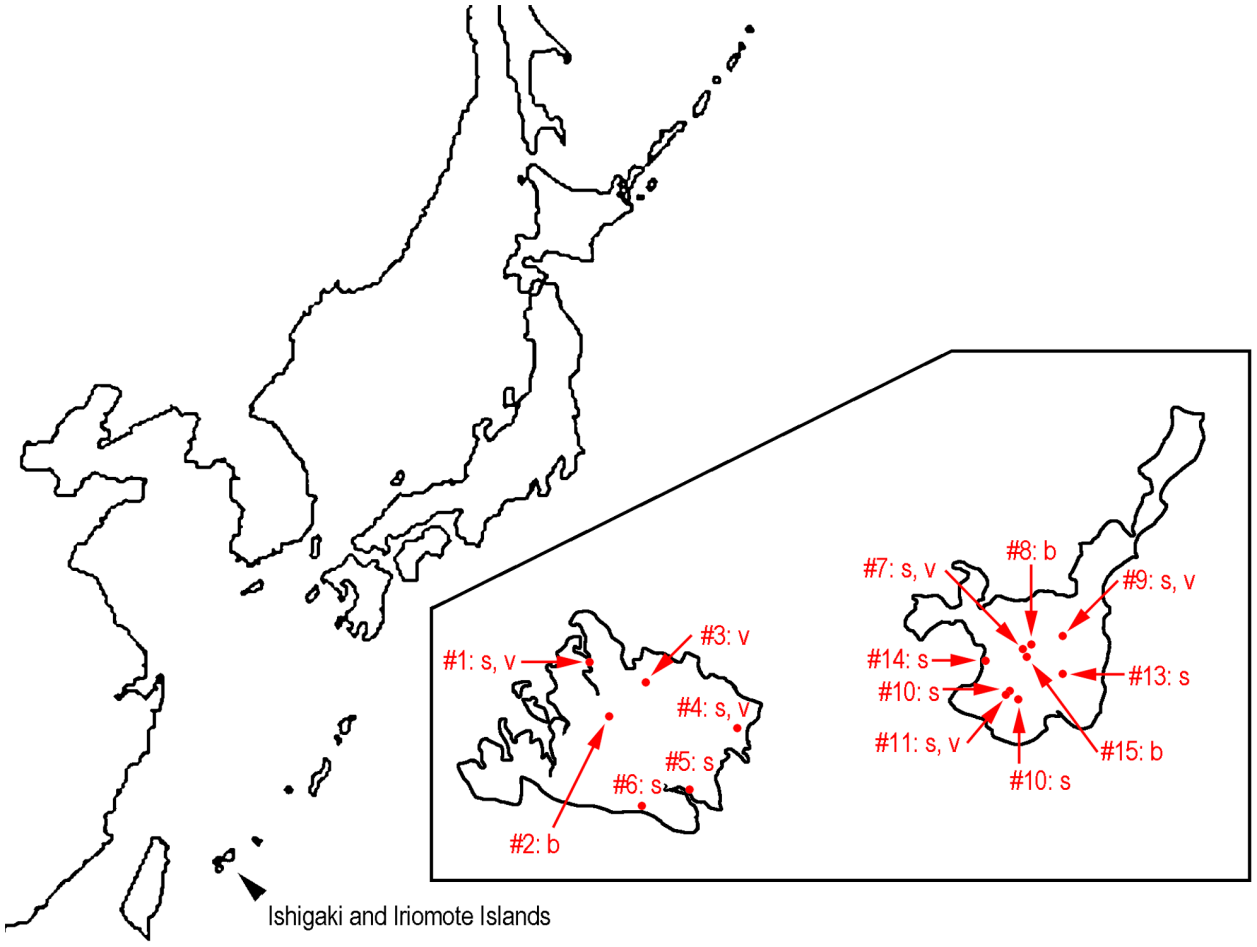
Outline map of collection localities for the samples examined in this study. For each locality and sampled fig species are suggested by abbreviations (b: *Ficus bengtensis*; s: *F. septica*; v: *F. variegata*).are listed. The GPS of the sites, sampled species and isolated nematode species (morphotype or genotype) \are summarized in Table S1.

No specific permissions were required for these locations/activities. Field studies did not involve endangered or protected species. The detailed location information is provided in Fig. 1 and supplemental information (Table S1).

A field survey of fig-associated nematodes was conducted during May to June, 2013 at the Ishigaki and Iriomote Islands, Okinawa, Japan. Various stages of fig syconia, i.e., unpollinated (young) and pollinated and developing (mature) ones, were collected from *F. variegata*, *F. septica* Burm. F. and *F. bengtensis* Merrill, and dissected on site using a portable dissecting microscope. The figs were pealed to remove the outer layers containing latex, and cut into small pieces in sterilized distilled water using a sterilized knife. Emerging nematodes were hand-picked with sterilized stainless needles for further analyses. They were heat-killed and fixed in TAF fixative (triethanolamine 2%, formalin 8%) for morphological specimens, or directly fixed in DESS [16] for further morphological and molecular profiling, and all materials were brought back to the laboratory. In addition to the materials collected on site, some syconia were brought back to the laboratory as back-up for additional sampling.

### Morphological observation

The TAF-fixed materials were examined under a dissecting microscope and separated into morphotypes. Each morphotype was processed using a glycerin–ethanol series with the modified Seinhorst's method [17], and mounted in glycerin according to the methods of Maeseneer and d'Herde [18]. The mounted specimens were designated as types, and used for morphometrics and morphological observations, micrographs and measurements. The male tail ventral view was observed using the glycerin-processed specimens with the methods provided in Kanzaki [19]. The morphological drawings and measurements (morphometrics) were conducted with the aid of a drawing tube connected to a Nikon Eclipse 80i (Nikon, Tokyo) facilitated with DIC optics. The micrographs were taken and edited with a digital camera system, DS-Ri1 (Nikon, Tokyo) and a computer program, Photoshop Elements v. 3 (Adobe, CA), respectively.

### Molecular profiles and phylogeny

For molecular analysis, DESS-fixed materials were washed and rehydrated in the sterilized distilled water, and observed using high magnification light microscopy to determine morphotypes. These observed specimens of *B. sycophilus* n. sp. were then transferred individually to 30 µl of nematode digestion buffer [20], [21] and digested at 60°C for 20 min., and the crude DNA solution was used for the PCR template. DNA base sequences of partial ribosomal DNA (ca 1.7-kb near-full-length small subunit [SSU] and 0.7-kb D2/D3 expansion segment of large subunit [D2/D3 LSU]) were determined for *B. sycophilus* n. sp. following the methods of Kanzaki and Futai [22] and Ye et al. [23].

The molecular phylogenetic status of *B. sycophilus* n. sp. was determined based upon SSU and D2/D3 LSU ribosomal RNA gene sequences using Bayesian, Maximum Likelihood (ML) and Maximum parsimony (MP) analyses. The SSU was compared with a wide-range aphelenchids and other infraorder species, and D2/D3 LSU, which is more suitable for lower level phylogenetic comparisons, was compared with those of closely related species. The species (operational taxonomic units: OTUs) compared with *B. sycophilus* n. sp. were determined according to the results of a BLAST homology search (http://blast.ncbi.nlm.nih.gov/Blast.cgi) and the OTUs used in the previous studies on aphelenchid phylogeny [24]–[27]. The species names and sequence accession numbers used in the present study were summarized in Table S2. Several tylenchid and panagrolaimid nematodes were used as outgroup species according to the previous studies [24]–[26]. The compared sequences were aligned using MAFFT [28], and the base substitution model was determined as GTR+I+G using MODELTEST version 3.7 [29] under the AIC model selection criterion. The Akaike-supported model, log likelihood (lnL), Akaike information criterion values, proportion of invariable sites, gamma distribution shape parameters, and substitution rates were used in the analyses. Bayesian analysis was performed using MrBayes 3.2 [30]; four chains were run for 4×10^6^ generations. Markov chains were sampled at intervals of 100 generations [31]. Two independent runs were performed, and after confirming the convergence of runs and discarding the first 2×10^6^ generations as ‘burn in’, the remaining topologies were used to generate a 50% majority-rule consensus tree. The PhyML 3.0 online version [32] was employed for the ML analysis. The analysis parameters obtained from the model selection procedure were adopted for the analysis, otherwise the default settings were used. The unweighted MP analysis was performed using PHYLIP 3.69 [33] with default settings. The tree topologies obtained from ML and MP analyses were evaluated with 1000 bootstrap pseudoreplications. The results obtained from Bayesian, ML and MP analyses were then compared to evaluate the phylogenetic position of the new species.

### Culturing attempt

Attempts were made to culture the nematode using the grey mold *Botrytis cinerea* Pers., a standard feeding resource fungus for mycophagous nematodes, and alfalfa callus (*Medicago sativa* L.), a standard feeding resource for plant-parasitic nematodes. The nematodes were extracted from the additionally-collected samples of *F. variegata* syconia, washed several times with sterilized distilled water, and transferred to fungal lawns on 2.0% malt extract agar (Difco malt extract: 2.0%; Agarose 2.0%) or alfalfa callus donated by Dr. T. Mizukubo (NARO Agricultural Research Center).

The transferred nematodes were kept at 23°C for a month, and were examined under a dissecting microscope every 5–10 days. Culturing attempts were replicated five times for both *B. cinerea* and alfalfa callus.

### Nomenclatural acts

The electronic edition of this article conforms to the requirements of the amended International Code of Zoological Nomenclature, and hence the new names contained herein are available under that Code from the electronic edition of this article. This published work and the nomenclatural acts it contains have been registered in ZooBank, the online registration system for the ICZN. The ZooBank LSIDs (Life Science Identifiers) can be resolved and the associated information viewed through any standard web browser by appending the LSID to the prefix “http://zoobank.org/”. The LSID for this publication is: urn:lsid:zoobank.org:pub:325625B8-D150-4836-B1A8-FCE3C8AC311C. The electronic edition of this work was published in a journal with an ISSN, and has been archived and is available from the following digital repositories: PubMed Central, LOCKSS.

## Results

*Bursaphelenchus sycophilus* Kanzaki, Tanaka, Giblin-Davis & Davies n. sp. urn:lsid:zoobank.org:act:6109FB02-E6FA-4570-8959-F918341319A9

### 

#### Type materials

The holotype male, nine paratype males and 10 paratype females were deposited in the United States Department of Agriculture Nematode Collection (USDANC), Beltsville, Maryland, USA, and 10 paratype males and 10 paratype females were deposited in the Forest Pathology Laboratory Collection, FFPRI, Tsukuba, Japan.

### Description

**Figure 2:**
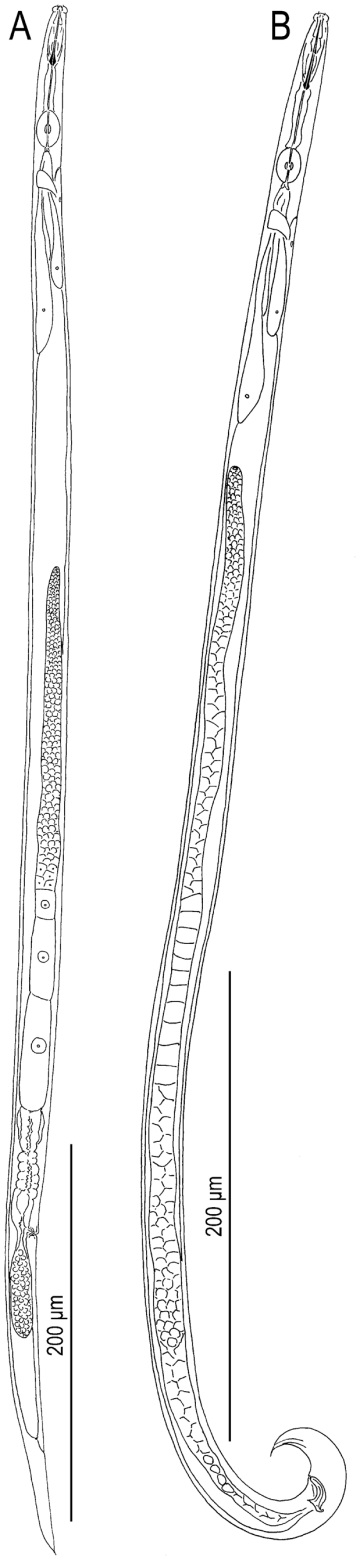
*Bursaphelenchus sycophilus* n. sp. A: Adult female; B: Adult male.

**Figure 3:**
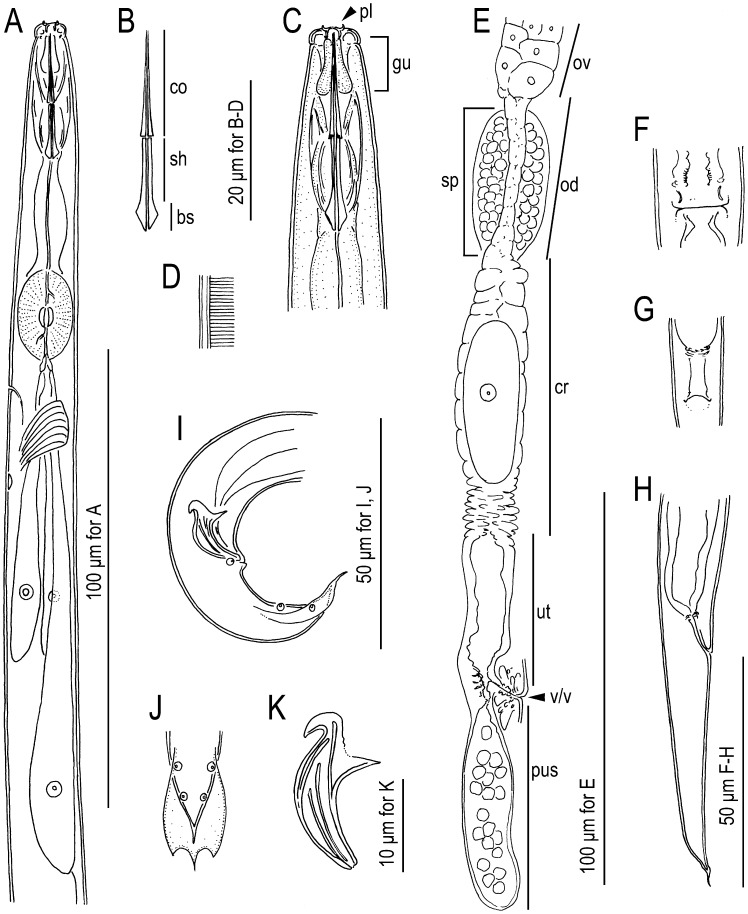
*Bursaphelenchus sycophilus* n. sp. A: Anterior region in left lateral view; B: Close-up of stylet (co: conus; sh: shaft; bs: basal swelling); C: Close-up of head region (pl: lip plate; gu: stylet guiding); D: Body surface pattern; E: Female reproductive system in right lateral view (ov: ovary; od: oviduct; sp: spermatheca or *receptaculum seminis*; cr: crustaformeria; ut: uterus; v/v: vagina and vulva; pus: post-uterine sac); F: Female vulval region in ventral view; G: Female anus and rectum in ventral view; H: Female tail in right lateral view; I: Male tail in right lateral view; J: Male tail tip (bursal flap) in ventral view; K: Male spicule in right lateral view.

**Figure 4:**
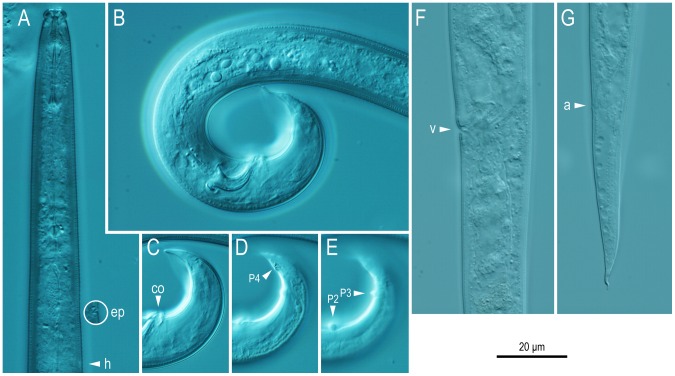
*Bursaphelenchus sycophilus* n. sp. A: Anterior region in right lateral view (ep: excretory pore encircled at the corresponding level of the body; h: hemizonid); B: Right lateral view of male tail; C–E: Right lateral view of male tail in different focal plane (co: cloacal opening; P2–P4: genital papillae); F: left lateral view of female vulval region (v: vulva); G: Left lateral view of female tail (a: anus).

Morphometric values are summarized in Table 1. Morphological illustrations and photographs are shown in Figs. 2–4.

**Table 1:** Morphometric values for *Bursaphelenchus sycophilus* n. sp.

	Male		Female
	Holotype	Paratypes	Paratypes
n	-	19	20
L	844	840±72 (738–964)	820±79 (666–933)
a	54.7	49.7±5.2 (39.0–59.3)	39.3±4.1 (32.6–45.2)
b	11.8	11.6±5.2 (9.7–13.0)	11.2±1.1 (9.0–13.0)
c	20.0	19.2±1.7 (15.7–22.7)	16.4±1.1 (14.5–18.7)
c′	2.9	2.9±0.3 (2.6–3.5)	4.8±0.7 (3.2–6.0)
T or V	67.2	67.4±5.1 (57.6–78.1)	79.2±1.2 (75.8–81.2)
M	51.9	52.4±2.5 (49.1–60.4)	53.2±1.6 (50–56.7)
Lip diam.	7.5	7.0±0.5 (6.0–7.5)	7.2±0.4 (6.5–8.0)
Lip height	3.5	3.7±0.4 (3.0–4.5)	3.7±0.4 (3.0–4.5)
Stylet conus length	13.9	14.2±1.0 (12.4–15.4)	15.5±0.9 (13.9–17.4)
Total stylet length	26.9	27.2±1.7 (23.9–29.4)	29.2±1.4 (26.7–32.8)
Median bulb length	16.4	17.0±1.5 (14.4–21.4)	17.6±1.5 (15.4–20.4)
Median bulb diam.	10.4	10.6±1.1 (9.5–13.4)	11.1±1.2 (9.5–13.4)
Median bulb length/diam.	1.57	1.61±0.2 (1.26–1.87)	1.59±0.1 (1.38–1.86)
Excretory pore from anterior end	86	87±6.0 (93–97)	85±5.7 (73–96)
Excretory pore from the base of median bulb	16.4	16.3±5.3 (14.4–21.4)	13.8±5.6 (3.0–21.9)
Nerve ring	87	88±3.4 (83–96)	87±3.8 (81–95)
Hemizonid from anterior end	97	100±5.6 (92–114)	97±4.3 (88–104)
Hemizonid from the base of median bulb	27.4	30.1±5.4 (23.9–41.8)	26.7±4.6 (17.9–35.3)
Gonad length (length from cloacal or vulval opening to anterior tip of gonad)	568	564±48 (494–659)	329±46 (198–389)
Cloacal/anal body diam.	14.4	15.0±1.4 (12.9–18.9)	10.6±1.5 (7.5–13.4)
Tail length	42	44±2.2 (40–49)	50±4.3 (43–57)
Spicule length (chord from anterior end of condylus to distal end)	15.6	16.0±0.8 (14.4–17.4)	-
Spicule length (curve from capitulum depression to distal end)	14.7	14.8±0.8 (12.9–15.9)	-
Post-uterine sac length	-	-	47±6.8 (34–60)
Post-uterine sac length per vulva-anus distance in %	-	-	39.3±4.7 (29.9–48.2)

All measurements are in µm in the form, average ± sd (range). The abbreviations for morphometric values are as follows. L: body length; a: body length/maximum body diameter; b: body length/length from anterior end to pharynx-intestine junction (ingestive organ length); c: body length/tail length; c′: tail length/cloacal or anal body diameter; T: testis length/body length in %; V: vulval position from anterior end in %; M: conus length to total stylet length in %.

#### Adults

Intermediate in body size, 738–964 µm and 666–933 µm in length for males and females, respectively. Body cylindrical, slender, weakly ventrally arcuate when killed by heat treatment. Male tail strongly recurved ventrally. Cuticle thin, finely annulated with a lateral field with four incisures. Lip region distinctly offset from body, separated from other body parts by a clear constriction, sub-rectangular or rounded, ca twice as broad as high in lateral view. A cuticular plate present at the anterior end. The edge of plate a little off-set, and appears like two cuticular projections in lateral view. Stylet very well-developed, separated into two parts: a conus occupying approximately 50% of the total stylet length and a shaft with a conspicuous and large basal swelling, but not forming a clear basal knob. Procorpus cylindrical, ca 1.5 metacorpal lengths ( = ca 1 stylet length) long, connected to a well-developed muscular oval-shaped metacorpus (median bulb) occupying ca 90% of the body diameter. Dorsal pharyngeal gland orifice opening into the lumen of the metacorpus midway between the anterior end of the metacorpal valve and the anterior end of the metacorpus. Metacorpal valve conspicuous, ca 1/5 of the metacorpal length, located at the centre or a little posterior to the centre of the metacorpus. Pharyngo-intestinal junction immediately posterior to the posterior end of the metacorpus. Pharyngeal glands well-developed. Dorsal pharyngeal gland ca 5–6 metacorpal lengths long, overlapping the intestine dorsally, ca 50% of the corresponding body diameter at the broadest part. Nerve ring surrounding pharyngeal glands and intestine ca 1 metacorpal length posterior to pharyngo-intestinal junction. Excretory pore visible, located near level of the nerve ring, i.e., varying between the posterior end of the metacorpus to ca 2 metacorpal lengths posterior to the metacorpus. Hemizonid ca 2 metacorpal lengths posterior to the metacorpus.

#### Male

Gonad single, outstretched in most individuals. Posterior 1/5 of gonad forms *vas deferens*, containing several well-developed sperm. Posterior end of *vas deferens* and intestine fused to form a narrow cloacal tube around the spicule. Sperm amoeboid, spermatocytes arranged in multiple (3–5) rows for anterior 1/5 of testis length, two rows for next 1/5, single row for middle part, with the well-developed sperm packed as 2–5 rows in the posterior part of testis. Tail region strongly arcuate ventrally, terminus claw-like in lateral view. Spicules paired, separate, mitten-shaped, stout, i.e., the length (chord from anterior end of condylus to cucullus) is ca 3 times the length of the widest part of the calomus–lamina complex: condylus short, rounded with anterior tip strongly recurved dorsally. Rostrum triangular, with pointed tip. Capitulum with clear depression immediately anterior to the anterior base of the rostrum. Lamina with two clear lines connecting the dorsal root of the condylus to the blunt tip, smoothly ventrally arcuate. Calomus–lamina complex widest at the posterior end of rostrum and calomus smoothly tapered along with lamina to distal tip. A cuticular limb present between calomus and lamina, extending from the middle of calomus to near the root of condylus. Connection between rostrum and calomus indistinctive, i.e., rostrum and calomus are connected with smooth curvature. Cucullus absent. Six (three pairs) genital papillae present: all are papilla-shaped, i.e., not gland-like (glandular papillae). First pair (P2) subventrally located, adcloacal, *i.e.*, at level of cloacal opening (CO). Second pair (P3) subventral ca 1/2 tail length posterior to CO. Third pair (P4) located ventrally ca 1/2 cloacal body diameter posterior to P3. Bursal flap present, covers the distal part from the level of P3, having oval shape with three projections at the posterior end. The middle projection longer and narrower than the others, appears as hair-like extension in the lateral view.

#### Female

Reproductive tract composed of ovary, oviduct, spermatheca, crustaformeria, uterus, vagina + vulva and post-uterine sac (branch). Ovary single, anteriorly outstretched, anterior end reflexed once in some individuals. Ovary constructed of flat, plate-like cells. Oocytes present in multiple (2–5) rows in anterior 1/2–4/5 of ovary with a couple of well-developed oocytes in a single row at the posterior end. Oviduct tube-like, constructed of large oval-shaped cells, connecting ovary and crustaformeria, sometimes occupied by well-developed oocytes. Spermatheca (*receptaculum seminis*) constructed of rounded cells, present as a branched overlapping of oviduct, i.e., branching out from anterior end of crustaformeria, slightly irregular oval shape, sometimes filled with well-developed sperm. Crustaformeria not conspicuous, formed of rather large, rounded cells. Uterus short with thick wall, sometimes containing a developing egg and several sperm. A sac-like expansion present on both sides of the uterus, which could be a part of the uterus. Dorsal uterine wall thickened at the uterus/vagina/post-uterine sac junction and a three-celled structure, where each cell has a cuticular (appears like fractal dots in LM observation) pronged structure, present at both right and left sides of the wall, but the structure is rather vague in fixed and mounted materials. Vagina slightly inclined anteriorly. Vulval opening lacking flap apparatus, both anterior and posterior vulval lips slightly expanded, and forming a dome-shaped slit in ventral view. Post-uterine sac conspicuous, filled with well-developed sperm in many individuals. Rectum present, seemingly functional, ca 1 anal body diameter (ABD) long, intestine–rectum junction constricted by sphincter muscle. Anus a small dome-shaped slit in ventral view; posterior anal lip slightly expanded in lateral view. Tail smoothly tapering to distal part, ca 3–6 ABD long. Tail tip region short, conical, with a hair-like projection.

#### Diagnosis

Besides the generic characters, *B. sycophilus* n. sp. is characterized by its unusually well-developed stylet, i.e., thick, long and possessing well-developed conus occupying ca 50% of total length and clearly developed basal swelling which forms a diamond-shape in lateral view, well-developed pharyngeal glands, three pairs of papilla-form male genital papillae and male spicule with strongly dorsally recurved condylus. The biological characters of inhabiting a fig syconium of *F. variegata* and being a plant parasite could also be considered diagnostic.

#### Relationship

Based on the male spicule morphology, i.e., the initial typological character of the genus [34], *B. sycophilus* n. sp. is similar to members of the “*B. eremus* group” (*B. eremus* (Rühm), *B. scolyti* Massey, *B. yongensis* Gu, Braasch, Burgermeister, Brandstetter & Zhang, *B. clavicauda* Kanzaki, Maehara & Masuya, *B. uncispicularis* Zhuo, Li, Li, Yu & Liao) and the “*B. leoni* group” (*B. eidmanni* (Rühm), *B. leoni* Baujard, *B. silvestris* (Lieutier & Laumond) and *B. borealis* Korentchenko) *sensu* Braasch et al. [34] and *B. maxbassiensis* (Massey). The new species and these species share the strongly dorsally arcuate condylus of the male spicule [35]–[44]. Within these 10 species, *B. maxbassiensis* is most similar to the new species, i.e., these two species share the extremely well-developed stylet [38], [41], [42]. However, the new species is distinguished from *B. maxbassiensis* by its lip morphology, with cuticular plate *vs* umbrella-like horizontal expansion, pharyngeal glands well-developed and extended vs. relatively short, position of excretory pore, posterior vs. anterior to median bulb, male bursal flap possessing three projections vs. rounded distal end, male spicule, the dorsal curvature in condylus is stronger in *B. sycophilus* n. sp., female post-uterine-branch occupying ca half of vulva-anus distance vs. occupying 2/3 or more of vulva-anus distance, female vulval structure, without any flap apparatus vs. slightly elongated anterior lip forming short flap and female tail, smoothly tapered with short conical distal end vs. smoothly tapered to distal end [38], [41], [42].

#### Molecular phylogeny

**Figure 5:**
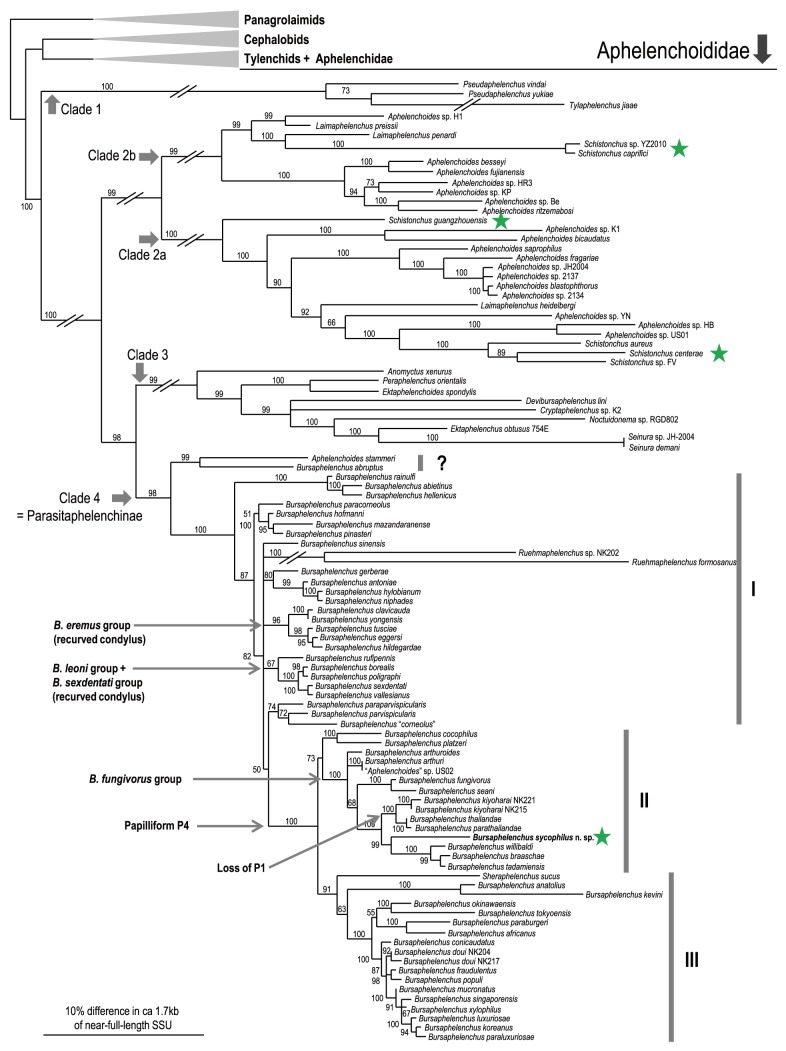
Molecular phylogenetic relationship among aphelenchid nematodes. The 10001st Bayesian tree inferred from near-full-length SSU under GTR+I+G model (lnL = 34563.4258; freqA = 0.2468; freqC = 0.1869; freqG = 0.2517; freqT = 0.3145; R(a) = 1.203; R(b) = 2.9772; R(c) = 1.1044; R(d) = 0.8365; R(e) = 4.1891; R(f) = 1; Pinva = 0.1959; Shape = 0.582). Posterior probability values exceeding 50% are given on appropriate clades. The blanch lengths for subfamily Parasitaphelenchinae which includes new species were expanded to show the topology clearly. The phylogenetic groups within the family Aphelenchoididae and within the subfamily Parasitaphelenchinae following Kanzaki et al. (2013) were indicated with thick arrows and bars, respectively. Parsimonious explanation on male genital papillae characters were indicated with thin arrows. The biological character, fig-association was indicated by stars.

**Figure 6:**
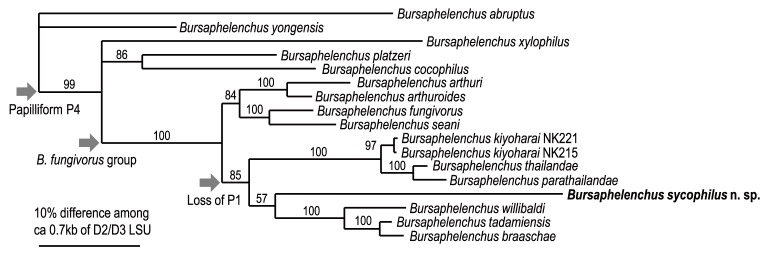
Molecular phylogenetic relationship among *Bursaphelenchus* nematodes belonging to ‘*B. fungivorus* group’. The 10001st Bayesian tree inferred from D2/D3 LSU under GTR+I+G model (lnL = 4855.5322; freqA = 0.1828; freqC = 0.1894; freqG = 0.3464; freqT = 0.2813; R(a) = 0.7168; R(b) = 2.1523; R(c) = 0.925; R(d) = 0.2398; R(e) = 4.6466; R(f) = 1; Pinvar = 0.322; Shape = ). Posterior probability values exceeding 50% are given on appropriate clades.

Because the tree topology and phylogenetic status of *B. sycophilus* n. sp. were consistent among analyses, only Bayesian trees are shown (Figs. 5, 6). In contrast to morphological similarity, *B. sycophilus* n. sp. phylogenetically belongs to clade II of the genus, and is close to *B. willibaldi* Schönfeld, Braasch & Burgermeister, *B. braaschae* Gu & Wang, *B. tadamiensis* Kanzaki, Taki, Masuya & Okabe, *B. kiyoharai* Kanzaki, Maehara, Aikawa, Masuya & Giblin-Davis, *B. thailandae* Braasch & Braasch-Bidasak and *B. parathailandae* Gu, Wang & Chen (Figs. 5, 6). These five species belong to the “*B. fungivorus* group” sensu Braasch et al. [34], and the group is characterized by the male spicule morphology possessing clear dorsal and ventral limbs Braasch et al. [34]. However, the new species is readily distinguished from members of the “*B. fungivorus* group” species based on the well-developed stylet and pharyngeal glands and male spicule morphology possessing a dorsally recurved condylus vs. a small condylus without dorsal curvature [34], [45]–[50].

#### Type host and locality

The type materials were obtained on June 6, 2013 from a syconium from a *Ficus variegata* tree planted on the Ishigaki Island, Okinawa, Japan (GPS: 24.41′12″N, 124.18′47″E, 59 m a.s.l).

#### Biological characters

The culturing attempts using *B. cinerea* and alfalfa callus were not successful. *Bursaphelenchus sycophilus* n. sp. showed no feeding behaviour on *B. cinerea* hyphae or on alfalfa callus parenchymal cells, and died out within one month of inoculation. Thus, the feeding preference (host range) of the nematode appears to be narrow, possibly specific to fig syconium tissue. Because a host-specific fig wasp species, *Ceratosolen appendiculatus* (Mayr), was the only insect found with the syconium from which type materials were obtained, the wasp is hypothesized to be the carrier (or host) insect of the nematode.

#### Etymology

The species was named after its characteristic habitat, the fig syconia.

## Discussion

The morphological characters and molecular phylogenetic status of *B. sycophilus* n. sp. are contradictory to each other, i.e., the new species shares a characteristic recurved condylus with members of the “*B. eremus* group”, “*B. leoni* group” and *B. maxbassiensis*, but is molecular phylogenetically close to members of the “*B. fungivorus* group” (Figs. 5, 6). Therefore, the recurved condylus found in the “*B. eremus* group”, the “*B. leoni* group” and the new species is considered as an analogous or convergent morphological character, i.e., the condylus morphology of the new species is a species-specific apomorphy.

**Figure 7:**
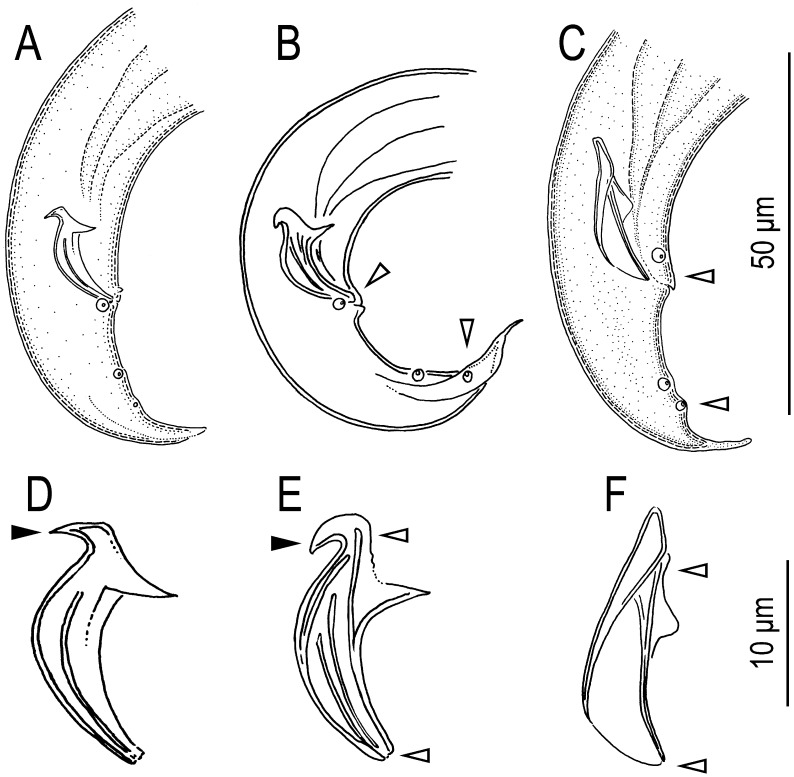
Comparison of male tail character among *Bursaphelenchus sycophilus* n. sp. ‘*B. eremus* group’ and ‘*B. fungivorus* group’. A: Male tail of *B. clavicauda* (*B. fungivorus* group) in right lateral view; B: Male tail of *B. sycophilus* n. sp. in right lateral view; C: Male tail of *B. tadamiensis* (“*B. fungivorus* group”) in right lateral view; D: Spicule of *B. clavicauda* in right lateral view; E: Spicule of *B. sycophilus* n. sp. in right lateral view; F: Spicule of *B. tadamiensis* in right lateral view. Convergent character (condylus) is suggested in black arrowhead and homologous characters (lack of P1 papilla, papilla-formed P4 papillae, presence of ventral limb and lack of cucullus) are suggested with white arrowhead.

Comparing the spicule and male tail morphology among members of the “*B. eremus* group”, the “*B. fungivorus* group” and *B. sycophilus* n. sp., three characters appear shared between the “*B. fungivorus* group” and *B. sycophilus* n. sp., i.e., the lack of a ventral single papilla anterior to cloacal opening (P1), lack of a cucullus on the spicule and possession of a ventral limb of the spicule (Fig. 7). The secondary loss of the P1 papilla is considered as an apomorphic character of some members of the “*B. fungivorus* group” [48], [49], and the presence of the ventral limb of the spicules is considered a shared character with members of the “*B. fungivorus* group” (Fig. 7). In addition to male tail morphology, *B. sycophilus* n. sp. and its close relatives are distinguished from the *B. eremus* group by the lack of a flap apparatus on the female vulva vs. possessing a short vulval flap (referred to as a side flap [51]) and the number of lateral lines being four vs. three [34], [52]. Thus, the presence of a ventral limb in the male spicule, papilla-shaped P4 papillae of males, lack of a ventral P1 papilla, and the lack of a female vulval flap are the synapomorphic characters shared by *B. sycophilus* n. sp. and its close relatives, i.e., the characters are congruent with the inferred molecular phylogenetic relationships.

The lip and stylet morphology of the new species is very unusual within the genus, and is similar to *B. maxbassiensis*, which was originally described as *Omemeea maxbassiensis* Massey because of its unusual lip and stylet morphology [41]. The large basal knobs are often found in the plant-parasitic tylenchids [53], and the extremely well-developed basal swellings of the stylet of the new species may be the result of convergent evolution of traits adaptive for plant parasitism in a fig sycone. Because the molecular phylogenetic status of *B. maxbassiensis* has not been established, we cannot determine whether their similarity is due to sharing a recent common ancestor or not. *Bursaphelenchus maxbassiensis* was isolated from the galleries of a bark beetle species, *Hylesinus californicus* (Swaine), infesting green ash, *Fraxinus pennsylvanica* Marsh. from North Dakota [41]. *Hylesinus californicus* sometimes bores into living trees [41], i.e., providing the nematode species with opportunities to encounter live plant tissue. Thus *B. maxbassiensis* may also have the ability to feed on live tree tissue. Re-isolation of *B. maxbassiensis* and detailed molecular and morphological comparison are necessary to examine the origin of plant parasitism of *B. sycophilus* n. sp.

The inferred molecular phylogenetic relationships and morphological characters are not congruent to each other in the subfamily Parasitaphelenchinae, which contains *Bursaphelenchus*, *Parasitaphelenchus* Fuchs, *Sheraphelenchus* Nickle and *Ruehmaphelenchus* Goodey [15]. Because of the morphological and biological diversity of *Bursaphelenchus*, the other three genera are included in the genus as subclades [24], [25], [54]. Therefore, a taxonomic revision is needed where all genera should be lumped together using their only common character, arrangement of the male genital papillae [15], or alternatively they should be separated into many small genera. If the new species and *B. maxbassiensis* shared a common ancestor, then the resurrection of the genus *Omemeea* Massey may be justified, if the genus (subfamily) is split into many small genera.

In the present study, culturing attempts using *B. cinerea* and alfalfa callus as food were not successful. The feeding resource preferences of several different nematodes, including *Bursaphelenchus* spp., have been previously studied, and in many cases, several different species of plant callus and ascomycete fungi, including *B. cinerea* were considered to be suitable food for aphelenchid nematodes [38], [55], [56]. Thus, although more replications using multiple species of fungi may be necessary, *B. sycophylus* n. sp. does not appear to feed on fungus, and the species is hypothesized to be an obligate plant parasite.

The obligate plant parasite, *B. cocophilus* can be cultured using fresh palm tissue [14], [57]–[59], although more detailed culturing attempts, e.g., using several different plant callus in including alfalfa, has not been conducted. A similar methodology, e.g., fig callus and/or fresh tissue of *Ficus variegata* may be available for the culture of *B. sycophilus* n. sp.

Several obligate and facultative plant parasites (pathogens) are known in the genus *Bursaphelenchus*, and they are phylogenetically distant from each other (Fig. 7), e.g., *B. xylophilus* (Steiner & Buhrer), the pathogen of pine wilt disease [60], *B. cocophilus* Cobb, the pathogen of red ring disease [57]–[59] and *B. sexdentati* Rühm, which has moderate to strong pathogenicity to pine trees [61]. This pattern of multiple lineages of plant parasitism may suggest the physiological plasticity of *Bursaphelenchus* nematodes in their feeding abilities, e.g., digestive enzymes. However, the morphology of the ingestive/digestive organs in these plant-parasites, including the obligate plant-parasite, *B. cocophilus* [62], are basically identical to that of other mycophagous *Bursaphelenchus* species [63]–[67]. The highly derived morphology of *B. sycophilus* n. sp. may represent the potential for morphological and developmental plasticity in the genus *Bursaphelenchus*.

Although *B. sycophilus* n. sp. clearly belongs to the genus *Bursaphelenchus*, its biological and morphological characters are similar to those of *Schistonchus* spp. The genus *Schistonchus* Cobb is known as to parasitize fig syconia, and are phoretically/parasitically associated with fig wasps [68]. Morphologically, the stylet of *Schistonchus* spp. is very similar to that of *B. sycophilus*, i.e., it has a long conus and distinct basal swellings. Interestingly, the genus is clearly paraphyletic [69]–[71] (Fig. 5). The similar morphology and life cycle of these fig-associated nematodes appears to have emerged from fungal feeding aphelenchoidid nematodes at least four times, inclusive of *B. sycophilus* n. sp. (Fig. 5).

Some biological characters of *B. sycophilus* n. sp. have not been clarified so far, e.g., insect interaction (parasitic or phoretic, and which developmental stage of nematode is carried by insect), detailed host range and distribution range. However, because the genotypes of *B. sycophilus* n. sp. isolated from several different locations on the Ishigaki and Iriomote Islands were identical, it is considered to be commonly distributed on these two islands. The distribution of *F. variegata* is widespread, from Ishigaki Island, Japan through South Eastern Asia to Northern Australia. However, regardless of multiple surveys of *F. variegata* in Northern Australia, *B. sycophilus* n. sp. has not been isolated from the area [69]. Therefore, the species is rare or absent from the region. To determine the distribution range of *B. sycophilus*, more surveys in South Eastern Asia and Northern Australia are needed.

In the present study, three species of figs were examined for their nematode association. The new species was isolated only from *F. variegata*, and was not found from the other two species, *F. septica* and *F. bengtensis*. Thus, *F. variegata* is considered as the specific fig host of *B. sycophilus* n. sp.

Center et al. [72] examined parasitized syconium tissue and suggested that each *Schistonchus* species has potential tissue specificity, which could lead to partitioning of the microhabitat inside the syconium. In the present study, detailed histological analysis was not conducted. Because *B. sycophilus* n. sp. often shares the same syconium with a currently undescribed *Schistonchus* species (Table S1), similar niche partitioning may be present contingent upon competition and other evolutionary pressure. Detailed analyses are necessary to clarify the biological interaction between *B. sycophilus* n. sp. and fig tissues, and *B. sycophilus* n. sp. and other fig-associated nematodes.

Because fig syconia represent an exclusive habitat relative to nematode immigration, i.e., only fig wasps and parasitic wasps enter the young figs, the history of multiple aphelenchoidid lineage introductions into different *Ficus* lineages is unclear. The simplest explanation might be through fig wasp host switching and lineage sorting for recent mixing. However, the convergence in feeding morphology between *B. sycophilus* n. sp. and the two or three different paraphyletic *Schistonchus* lineages suggest other possible scenarios, such as rogue introductions of fungal feeding nematodes by other fig-associating insects or other unknown aspects concerning fig biology. For example, the new species is close to the species associated with ambrosia beetles (*B. kiyoharai*) [48] and stag beetles (*B. tadamiensis*) [49], and some ambrosia beetles invade the petiole of living trees in tropical region [73], [74]. Given the relatively short branch length (genetic distance) between *B. sycophilus* n. sp. and its related *Bursaphelenchus* spp., the new species seems to have adapted to this closed environment relatively rapidly. More detailed analyses, e.g., comparative genomic analyses using the new species and its close relatives, may yield interesting information concerning the genes involved in switching feeding habitats and the development of plant parasitism.

## Supporting Information

Table S1 **GPS coordinates of sampling sites and nematode species (morphotypes and/or genotypes) isolated from the materials.**
(XLS)

Table S2 **Accession numbers and species names of nematodes used in the phylogenetic analysis.**
(XLS)
